# Editorial: Evidence-based strength intervention in multiple contexts

**DOI:** 10.3389/fpsyg.2022.1081610

**Published:** 2022-11-11

**Authors:** Xixi Sun, Wenjie Duan

**Affiliations:** School of Social and Public Administration, East China University of Science and Technology, Shanghai, China

**Keywords:** evidence-based practice, strength-based perspective, positive psychology, positive interventions, strength interventions

## Introduction

Evidence-based practice (EBP) is a process of making practice decisions and evaluating effectiveness through identifying, selecting, and applying the best scientific evidence (Rubin, 2008; Nevo and Slonim-Nevo, 2011; Kagan, 2022). Randomized control trials (RCTs) are considered one of the strongest evidence and the gold standard methodology with its internal validity in detecting a causal relationship between treatment and outcome and measuring the effectiveness of a treatment (Sibbald and Roland, 1998). A few research including RCTs, has proved that EBP can bring about positive outcomes (Stanhope et al., 2010) and has spread to wider areas, including but not limited to psychology, psychiatry, public health, and social work (APA Presidential Task Force on Evidence-Based Practice, 2006; Brownson et al., 2009; Gambrill, 2011).

Scholars in these health-related fields have accumulated a broad range of research on science-based health promotion programs, which paid more attention to repairing the weakness or problems of individuals and communities. However, it has been recognized that the traditional approach is insufficient or inefficient enough to help individuals and communities achieve sustained outcomes since the emergence of positive psychology in the United States about two decades ago (Gable and Haidt, 2005). Positive psychology focuses on the scientific study of positive experience, positive individual traits, and environmental strength (Duckworth et al., 2005) and views human life from a positive perspective with a central mission to identify, develop, and evaluate interventions that aim to enhance wellbeing (Carr et al., 2020). Compelling evidence illustrated that positive emotion represents a separate psychological process which distinct from negative emotion (Fredrickson, 1998; Duckworth et al., 2005). The understanding of the scope of health is therefore broadened from removing ill-being to being and living well (Neuhaus et al., 2022). In other words, the interventions should not only be designed to help at-risk populations get back to normal life but also to help at-normal populations to a better life.

Although positive psychology is a relatively young branch of psychology, a few strengths-based intervention studies were conducted (Gander et al., 2012; Duan et al., 2018; Bu and Duan, 2019), and the number of publications was increased in recent years. Part of the existing studies demonstrated the effectiveness of strengths-based interventions (Duan et al., 2013, 2022b; Carr et al., 2020), while others constructed and examined the validation of character strengths-based interventions (Niemiec, 2018; Duan et al., 2022a). However, research on how and why these strengths-based interventions work remains unclear (Ghielen et al., 2017). To address these questions, we collected a series of articles to represent the latest empirical study on evidence-based strengths interventions in multiple contexts, including psychology, psychiatry, public health, and social work backgrounds. We believe such work will be critical in integrating personal and environmental strengths to foster wellbeing in different settings, including but not limited to clinical, non-clinical, community, and educational settings and across treatment, prevention and promotion models. Furthermore, a deeper understanding of underlying mechanisms of change present in the situations will be attained and can be used to innovate interventions. In this Research Topic, 16 works were collected and published in three journals (i.e., *Frontier in Psychology, Frontier in Psychiatry*, and *Frontier in Public Health*), illustrating a snapshot of the latest progress of evidence-based strength interventions.

## Evidence-based strength intervention in the psychology context

This section contains nine articles investigating producing positive psychological, social, cultural, and health-based outcomes. Three experimental studies were collected in this section, including one RCT and three quasi-experiment studies. A three-group RCT designed by Lai et al. provided the first evidence of the effectiveness of probation service and the additional use of a positive family holistic health intervention. Results showed that the intervention integrating with positive psychology themes enhanced probationers' holistic health, family communication, and their relationships with probation officers. Saracostti et al. designed a quasi-experiment trial to prove that the Ecological, Participatory, Integral, and Contextualized Family-School Collaboration Model positively influences home-based involvement, memory, attention, and intrapersonal skills in the first cycle of elementary education. Tao et al. used a pretest-posttest method of quasi-experimental design to examine the impact of forgiveness interventions. It is revealed that the forgiveness intervention can effectively improve the positive mental strength (i.e., forgiveness, empathy, and harmony) of adolescents with high levels of trait anger. Another quasi-experimental trial was conducted by Corbu et al. to test the effect of a Positive Psychological Micro-Coaching program on non-executive workers' psychological capital. Results implicated that short-term positive psychological coaching is a valuable way to develop personal resources in improving goal achievement and then work-related goals in non-executive employees.

Two studies conducted mediation analysis among older adults in the Chinese context, and one study conducted content analysis among Chinese female students in the United Kingdom. Cheng et al. investigated the impact of objective isolation and subjective social isolation on the mental health of older Chinese adults and the mediating effect of aging attitudes. Using the sample from the 2014 Chinese Longitudinal Aging Social Survey, the research showed that aging attitudes play a significant mediating role between social isolation and mental health. Yang et al. used a sample from the 2013 Chinese General Social Survey to examine the relationship between life satisfaction and lifestyle, the number of children, and widowhood status. The established moderated mediation model illustrated that lifestyle partly mediated the relationship between widowhood and life satisfaction while the number of children moderated the relationship between widowhood and lifestyle and between lifestyle and life satisfaction. Zhang and Tang's qualitative study explored factors that impact Chinese students' choice of study destination and choice of subject and program. It is shown that cultural capital, gender, class, and family involvement all influenced Chinese female students' aspiration to study in the United Kingdom, and despite the fact these students have the privilege to study abroad, female students from the middle class are constrained by Chinese gender norms and class background when making educational choices.

Two studies in this section provided evidence-based strength intervention with reliable and valid methods of assessment tools. Duan et al.'s study was the first to examine the factor structure of the Physical Disability Resilience Scale (PDRS) in the Chinese context based on the Multiple Sclerosis Resiliency Scale. The revised PDRS with four subscales (i.e., Emotional and Cognitive Strategies, Physical Activity and Diet, Peer Support, and Support from Family and Friends) showed good reliability and validity in assessing resilience among Chinese people with a physical disability. To et al. developed the Parent Empowerment *via* Transformative Learning Questionnaire (PETLQ) and confirmed it as a scale with sufficient factorial validity and internal consistency for assessing parents' attitudes and competence in parent empowerment and for evaluating the effectiveness of parenting intervention programs. It is notable that even though these two studies were classified in psychology contexts, social workers were involved in the research process as key members.

## Evidence-based strength intervention in the psychiatry context

Three articles were included in this section with a psychiatric focus on evidence-based strength intervention, with two systematic review articles and one correlation study. Two systematic reviews summarized six strategies used to improve community services for deinstitutionalized patients with severe mental disorders (Fulone et al.) and 117 different coaching tools (18 overarching coaching techniques) used in the different phases of the Positive Psychological Coaching model (Richter et al.). To evaluate the mediatory role of sense of coherence, Hori et al. conducted a survey among healthcare professionals in a Japanese general hospital and found that sense of coherence mediated the relations between empathy and both self-vigor mood and self-depression mood. This research indicates that more effective empathy performance interventions need to be developed for healthcare professionals.

## Evidence-based strength intervention in the public health context

In the public health context, researchers paid attention to the wellbeing of patients with physical or mental health issues and the fidelity of nurses-delivered healthcare programs, including four articles.

Three articles are systematic reviews studying the wellbeing of patients. Gao et al. conducted a network meta-analysis to examine the safety and effectiveness of surgical interventions for pure cervical radiculopathy. This research illustrates that all surgical interventions can achieve satisfactory results and surgeons can choose appropriate surgical interventions based on their strengths and patient-related factors. Zhao et al.'s systematic review of RCTs on the effects of the tourniquet on pain and return to function showed that the routine use of a tourniquet during total knee arthroplasty was not recommended due to more pain, slower functional recovery, and more complications. Another systematic review and meta-analysis conducted by Zhang et al. found that horticultural therapy had a significant positive effect on depressive symptom reductions in the elderly. To enable the systematic evaluation of parenting program delivery and to better identify the therapeutic components that enable targeted efforts at improvement, Anis et al. developed a fidelity assessment checklist to make the program-delivery evidence-based.

## Future research

The collection of this Research Topic presents the features of the broadening scope and high level of evidence (APA Presidential Task Force on Evidence-Based Practice, 2006; Thyer and Pignotti, 2011; Lomas et al., 2020). Regarding broadening scope, 16 articles looked deeply and critically at different groups, including adolescents, families, the elderly, patients, workers, and healthcare professionals. Beyond the primary focus on the individual person, these articles moved toward more contextually-oriented and system-informed approaches, looking into multiple interpersonal and ecological factors that might create nurturing environments and positive institutions (Lomas et al., 2020). The broadening scope was also reflected in the inclusion of cross-cultural research. It is valuable to test tools, constructs, and methodologies across populations that developed in the Western context, illustrating how culture influences people's understanding and experience of the world (Lomas et al., 2020). The high quality of these studies can be seen from the fact that more than half of the research were systematic reviews, RCTs, and quasi-experiment studies, contributing to the high level of evidence.

Despite the progress made through these studies, more work is needed further to develop positive psychology in the evidence-based strength intervention field. It is worth noticing that this Research Topic had not attracted any study from social work, and the number of articles from the psychiatric and public health field was much smaller than psychology. Thus, from the evidence-based perspective, there is still a need for more high-quality empirical evidence to provide a theoretical rationale for the exploration in broadening scope and the establishment of a stronger and broader evidence base, especially from but not limited to social work, public health, and psychiatry professional perspectives. From the strength-based perspective, the current research still mainly focused on individual-level phenomena and did not deeply explore contextual factors. Future research might focus more on contextual and structural factors that impact personal, group, and communal wellbeing in multiple contexts and explore more mediators and moderators that can explain the effectiveness of evidence-based strengths interventions and the underlying mechanisms of positive changes.

## Author contributions

XS wrote the draft of this editorial. WD finalized the version and submitted it to the journal. All authors contributed to the article and approved the submitted version.

## Funding

The East China University of Science and Technology Supporting Funds for Scientific Research Strengths-Based Interventions in Multiple Contexts.

## Conflict of interest

The authors declare that the research was conducted in the absence of any commercial or financial relationships that could be construed as a potential conflict of interest.

## Publisher's note

All claims expressed in this article are solely those of the authors and do not necessarily represent those of their affiliated organizations, or those of the publisher, the editors and the reviewers. Any product that may be evaluated in this article, or claim that may be made by its manufacturer, is not guaranteed or endorsed by the publisher.
